# Recent Advances in Cancer Immunotherapy

**DOI:** 10.3390/biom11020335

**Published:** 2021-02-23

**Authors:** Kenichi Suda

**Affiliations:** Division of Thoracic Surgery, Department of Surgery, Kindai University Faculty of Medicine, Osaka-Sayama 589-8511, Japan; ascaris@surg2.med.kyushu-u.ac.jp; Tel.: +81-72-366-0221

The strategy to use the immune system to fight cancer is not a novel concept; in 1891, Coley reported the treatment of three cases of sarcoma by inoculation with erysipelas [1]. However, less than 10 years have passed since cancer immunotherapy began attracting a great deal of attention from clinicians, oncologists, and cancer researchers.

In 2011, ipilimumab, an immune checkpoint inhibitor (ICI) that blocks CTLA-4, was the first ICI approved by the US FDA and used to treat patients with late-stage melanoma. This was rapidly followed by the development of monoclonal antibodies targeting another immune checkpoint molecule, PD-1, and its ligand PD-L1 [2]. These anti-PD-1 (nivolumab and pembrolizumab) and anti-PD-L1 agents (atezolizumab, durvalumab, and avelumab) have shown dramatic and durable response in clinical trials and have now become some of the most widely prescribed anticancer drugs for a wide range of malignancies including non-small cell lung cancer, melanoma, cutaneous squamous cell carcinoma, urothelial carcinoma, cervical cancer, Hodgkin’s lymphoma, head and neck squamous cell carcinoma, Merkel cell carcinoma, renal cell carcinoma, MSI-H or dMMR colorectal cancer, and hepatocellular carcinoma [3]. Furthermore, chimeric antigen receptor (CAR) T cell therapies have recently joined the list of approved cancer immunotherapies as treatments for mantle cell lymphoma or certain types of large B-cell lymphoma.

The dramatic development and evolution in cancer immunotherapies has accelerated basic, translational, and clinical research in this field. The purposes of these research studies include, but are not limited to, the following areas: (i) evaluating the immunological status of cancer/the tumor microenvironment, (ii) finding biomarkers to predict the efficacies of current immunotherapies, (iii) exploring inherent and/or acquired resistance mechanisms, and (iv) developing novel immunotherapies. Because cancer immunotherapies are active against a wide range of malignancies, the findings obtained in the analysis of one type of cancer may be applicable to other malignancies. Therefore, in this Special Issue, I set the scope to provide a broad (i.e., pan-cancer) and updated overview on the recent progress of all types of cancer immunotherapy research. The collection includes two original research papers and four well-summarized review articles.

Among these six papers, three are classified into topic (i) evaluating the immunological status of cancer/the tumor microenvironment. The first study is from our group, which reported that PD-L1 expression is significantly lower in lung adenocarcinomas with ground-glass opacity (GGO) compared with those without GGO (pure-solid tumors) [4]. This study reveals the immunological differences between these two types of lung adenocarcinomas. Additionally, the results of this report provided an important speculation regarding the prognostic impact of PD-L1 expression in lung adenocarcinomas, which had been controversial in previous studies [5]; the ratio of tumors with GGO, which are associated with favorable prognosis, will determine the apparent prognostic implication of PD-L1 expression in a cohort [4]. The second paper on this topic is a review article written by Fujimura et al. [6]. The authors comprehensively reviewed the roles of immunosuppressive cells, such as tumor-associated macrophages, myeloid-derived suppressor cells, regulatory T cells, and tumor-associated neutrophils in the development and maintenance of melanoma and non-melanoma skin cancers. The third study is a review paper written by Baleydier et al., which summarized a difficult but interesting issue: cancer immunotherapies for children with cancer with primary immunodeficiencies [7]. First, the authors summarized the list of primary immunodeficiencies prone to cancer (lymphoma or other hematological malignancies in most of the cases) and their corresponding gene defects (see Table 1 in their review) [7]. The authors then described the molecular mechanisms of immune deficiencies in each syndrome and the oncogenic mechanisms involved in tumorigenesis in children with primary immunodeficiencies. The authors finally summarized the possibilities of cancer immunotherapies, which were classified into humanized monoclonal antibodies, cell therapies including CAR T cells, and immunomodulators, for this specific cohort.

The second topic, (ii) biomarkers to predict the efficacies of current immunotherapies, is one of the hottest issues in the field of cancer immunotherapy research. PD-L1 expression, which is the only approved companion/complimentary diagnostic for the efficacy of anti-PD-1/PD-L1 agents, and other potential biomarkers, such as tumor mutation burden or infiltration of CD8 positive T cells, are all imperfect in predicting the efficacies of cancer immunotherapies. The paper by Fujimura et al. further highlights this point, stating that immunosuppressive cells are also the target of current immunotherapies and that tumor-associated macrophage-related biomarkers, such as soluble CD163 or CXCL5, will be useful to predict the efficacy of anti-PD-1 agents in melanoma [6].

(iii) Exploration for the inherent and acquired resistance mechanisms to cancer immunotherapies is also an important topic. In this Special Issue, two groups independently summarized the resistance mechanisms to checkpoint inhibitors in solid tumors [3,8]. Koustas et al. summarized the pan-cancer data for the resistance mechanisms to ICIs that include roles of the tumor microenvironment and autophagy, as well as genetic and epigenetic alterations (such as PTEN loss, JAK1/2 mutations, or microphthalmia-associated transcription factor suppression). The authors also summarized the clinical trials for many types of malignancies that are evaluating the combined immunotherapies to overcome ICI resistance (see Table 1 in their study) [8]. The second paper, written by Perrier et al., focused on the epigenetic changes (modifications of histones, DNA methylation, and miRNAs) that are related to ICI resistance. The authors also provide a useful summary of miRNAs that regulate PD-L1 expression in many types of cancer cells (see Table 2 in their study) [3].

Regarding topic (iv) developing novel immunotherapies, there is only one research article on this area in the Special Issue. In this study, Moz et al. reported that treatment with the vitamin D analogue calcipotriol prevented apoptosis signaling of peripheral blood mononuclear cells that is induced by pancreatic ductal adenocarcinoma cells [9].

The roles and impacts of cancer immunotherapies will expand dramatically, including through the development of immunotherapies for special populations, immunotherapy combinations, or immunotherapies in neoadjuvant or adjuvant settings. I expect that thematic issues for pan-cancer immunotherapies, as exemplified by this Special Issue, will accelerate the understanding of the immunological status of cancer/the tumor microenvironment, the exploration of novel predictive biomarkers for immunotherapies, the development of strategies to overcome inherent/acquired resistance to immunotherapies, and the clinical application of novel immunotherapies in the near future.
